# Harnessing amino acid pathways to influence myeloid cell function in tumor immunity

**DOI:** 10.1186/s10020-025-01099-4

**Published:** 2025-02-04

**Authors:** Jiongli Pan, Yi Lin, Xinyuan Liu, Xiaozhen Zhang, Tingbo Liang, Xueli Bai

**Affiliations:** 1 Department of Hepatobiliary and Pancreatic Surgery, The First Affiliated Hospital, School of Medicine, Zhejiang University, Hangzhou, Zhejiang China; 2 Zhejiang Provincial Key Laboratory of Pancreatic Disease, The First Affiliated Hospital, School of Medicine, Zhejiang University, Hangzhou, Zhejiang China; 3 Health Science Center, Ningbo University, Ningbo, China

**Keywords:** Amino acid metabolism, Myeloid cells, Immunometabolism, Metabolic reprogramming, Cancer immunotherapy

## Abstract

Amino acids are pivotal regulators of immune cell metabolism, signaling pathways, and gene expression. In myeloid cells, these processes underlie their functional plasticity, enabling shifts between pro-inflammatory, anti-inflammatory, pro-tumor, and anti-tumor activities. Within the tumor microenvironment, amino acid metabolism plays a crucial role in mediating the immunosuppressive functions of myeloid cells, contributing to tumor progression. This review delves into the mechanisms by which specific amino acids—glutamine, serine, arginine, and tryptophan—regulate myeloid cell function and polarization. Furthermore, we explore the therapeutic potential of targeting amino acid metabolism to enhance anti-tumor immunity, offering insights into novel strategies for cancer treatment.

## Background

Amino acids are essential biological macromolecules that play crucial roles beyond their traditional function as building blocks for proteins. They are deeply involved in a wide array of cellular processes, including metabolism, signal transduction, and gene expression. In immune cells, amino acids regulate activation and function through various mechanisms, such as providing energy, maintaining redox balance, and participating in epigenetic and post-translational modifications (Kelly and Pearce 2020).

Myeloid cells, which originate from hematopoietic stem cells in the bone marrow, comprise a diverse group of immune cells, including macrophages, dendritic cells (DCs), neutrophils, and myeloid-derived suppressor cells (MDSCs). Each of these cell types performs distinct functions. Macrophages are classified into two major subsets: M1, which promotes inflammation, and M2, which exhibits anti-inflammatory properties. M1 macrophages release inflammatory mediators such as interleukin-1β (IL-1β), tumor necrosis factor-α (TNF-α), and nitric oxide (NO) to eliminate pathogens. In contrast, M2 macrophages secrete anti-inflammatory cytokines like IL-10 and transforming growth factor-β (TGF-β) to resolve inflammation and promote tissue repair (Anderson et al. 2021; Locati et al. 2020). Recently, researchers have proposed a new macrophage phenotype, M3, which is considered either an intermediate state in the polarization process toward M1/M2-like phenotypes (Jackaman et al. 2016) or a transitional phenotype representing the shift between M1 and M2 states (Malyshev and Malyshev 2015). DCs, as primary antigen-presenting cells, activate T cells by presenting antigens. Additionally, DCs can promote tolerogenic immune responses by inducing regulatory T (Treg) cells, thereby preventing excessive immune reactions (Heras-Murillo et al. 2024). Neutrophils, the most abundant myeloid cells in peripheral blood, play a crucial role in combating pathogens through phagocytosis, degranulation, and the release of neutrophil extracellular traps (NETs) (Burn et al. 2021). MDSCs are immature myeloid cells that are categorized into two major groups: polymorphonuclear MDSCs (PMN-MDSCs, also known as granulocytic-MDSCs) and monocytic MDSCs (M-MDSCs) (Bronte et al. 2016). MDSCs promote tumor progression by depleting essential nutrients and releasing inhibitory cytokines that suppress immune cell function (Yang et al. 2020).

Myeloid cell functions are characterized by their plasticity, enabling them to perform distinct roles in varying environments (Locati et al. 2020; Tcyganov et al. 2018; Galli et al. 2011). During pathogen invasion, M1 macrophages, DCs, and neutrophils are involved in pathogen clearance, while M2 macrophages facilitate the resolution of inflammation. In the tumor microenvironment, however, tumor cells can drive myeloid cells to adopt an immunosuppressive phenotype through various mechanisms, such as the release of cytokines or depletion of nutrients, thereby promoting tumor progression. Understanding the mechanisms underlying the phenotypic and functional transitions of myeloid cells is critical for controlling inflammation and developing cancer therapies.

The functional diversity of myeloid cells is largely regulated by metabolic reprogramming. For instance, M1 macrophages predominantly rely on glycolysis and the pentose phosphate pathway (PPP) to meet their energy demands, while M2 macrophages enhance fatty acid oxidation (FAO) and oxidative phosphorylation (OXPHOS) to execute their functions (Viola et al. 2019). Recent studies have highlighted the role of amino acid metabolism in coordinating myeloid cell functions. For example, serine promotes M1 macrophage polarization (Rodriguez et al. 2019), while glutamine facilitates M2 macrophage polarization (Liu et al. 2017). In the tumor microenvironment, immunosuppressive myeloid cells upregulate amino acid-metabolizing enzymes, such as Arginase 1 (Arg1) and Indoleamine 2,3-dioxygenase 1 (IDO1), to deplete arginine and tryptophan—amino acids essential for T cell activation and function—thus promoting tumor progression.

Given the significant impact of amino acid metabolism on myeloid cell functions, numerous inhibitors targeting key enzymes in these metabolic pathways have been developed and tested in clinical trials. These therapeutic agents aim to modulate the tumor microenvironment by altering the metabolic profiles of myeloid cells, thereby inhibiting their consumption of amino acids and restoring T cell function while inducing a shift in myeloid cell phenotype. The promising results from these trials underscore the potential of targeting amino acid metabolism as a novel therapeutic strategy in cancer treatment.

In this review, we provide a comprehensive overview of the latest research on the role of amino acid metabolism in myeloid cell functions. We focus on widely studied amino acids such as glutamine, serine, arginine, and tryptophan. We first introduce the metabolic pathways of these amino acids and then discuss how they regulate the phenotypic transitions of myeloid cells and mediate immunosuppressive functions. Additionally, we examine the role of amino acid sensors, including general control nonderepressible 2 (GCN2) and mechanistic target of rapamycin complex 1 (mTORC1), in modulating these processes. Furthermore, we explore emerging clinical evidence supporting the therapeutic potential of targeting amino acid metabolism to modulate immune responses and improve patient outcomes. This review aims to illuminate the critical intersection between metabolism and immunity, offering insights into the development of novel therapeutic strategies.

## Amino acid metabolism: pathways and mechanisms

### Amino acid transport and uptake

Amino acid transporters facilitate the transport of amino acids into or out of cells and organelles. Based on their transport characteristics and substrates, amino acid transporters can be categorized into several families, such as systems A, N, L, X^-^_C_ (Kandasamy et al. 2018). It is important to note that a single transporter may not exclusively transport one type of amino acid, and conversely, a single amino acid can be transported by multiple transporters. For example, SLC1A4 can transport alanine, serine, cysteine, threonine, glutamine, but primarily transports alanine and serine. Glutamine can be transported by SLC1A5, SLC6A14 and SLC38A1. These properties add to the complexity of studying amino acid transporters. Some amino acids are exchanged by heterodimeric amino acid exchangers. For instance, SLC7A11 (xCT) forms a dimer with SLC3A2 (CD98 heavy chain or CD98hc), mediating the uptake of cystine and the exit of glutamate. Similarly, SLC7A5 (LAT1) forms a dimer with SLC3A2, facilitating the transport of leucine, isoleucine, and valine.

### Sensing mechanisms of amino acids

GCN2 and mTORC1 are two intracellular kinases that play crucial roles in sensing amino acid levels within cells. Specifically, GCN2 is activated in response to amino acid deprivation, signaling a lack of essential nutrients, while mTORC1 is activated by elevated amino acid levels, indicating nutrient abundance.

**Fig. 1:**
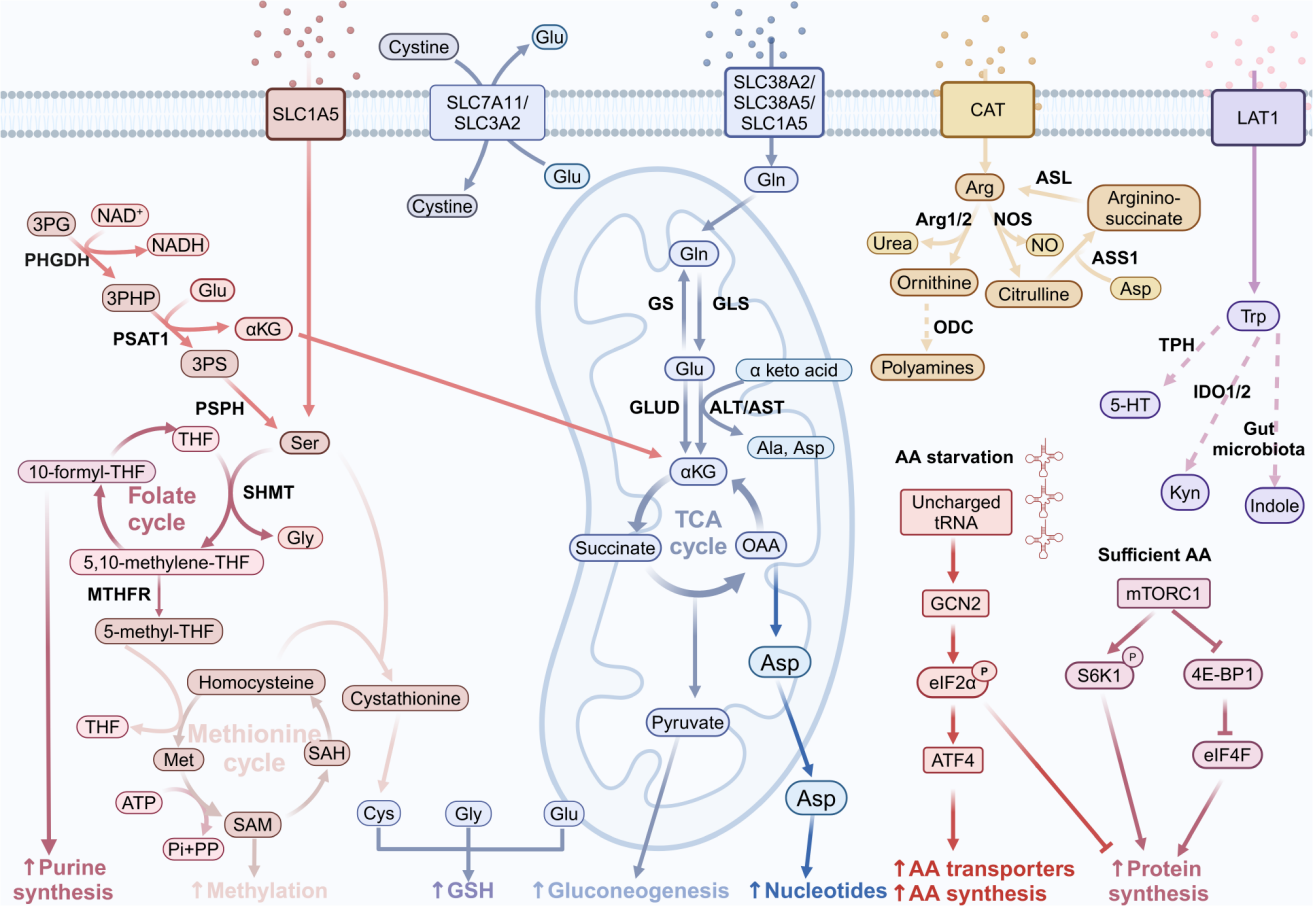
The overview of amino acid metabolism

In conditions of amino acid abundance, amino acid-charged tRNAs provide the necessary substrates for protein translation. However, during amino acid starvation, uncharged tRNAs accumulate, leading to the activation of GCN2 (Dong et al. 2000). This activation results in the phosphorylation of eIF2α, which inhibits its role in the translation process, thereby halting the general translation of non-essential proteins (Zhao et al. 2023). Additionally, the phosphorylation of eIF2α induces the translation of ATF4 mRNA. ATF4, a transcription factor, binds to the promoters of specific genes and activates their transcription, including those involved in amino acid transport and synthesis (Kilberg et al. 2009) (see Fig. 1). Consequently, GCN2 conserves cellular resources during periods of amino acid scarcity.

mTORC1 is a serine/threonine kinase that integrates environmental and intracellular cues to regulate cell growth. Under conditions of sufficient intracellular amino acids, the Rag GTPases dimer, in conjunction with the Ragulator complex, facilitates the translocation of mTORC1 to the lysosomal membrane (Sancak et al. 2010). The lysosomal membrane is enriched with the mTORC1 activator Rheb, which subsequently activates mTORC1. Conversely, the TSC complex hydrolyzes the GTP bound to Rheb, leading to Rheb inactivation and, thus, the inhibition of mTORC1 activity (Liu and Sabatini 2020).

Upon activation, mTORC1 phosphorylates ribosomal protein S6 kinase 1 (S6K1) and 4E-binding protein-1 (4EBP1). The phosphorylation of S6K1 enhances the synthesis of proteins and nucleotides. Additionally, 4EBP1, an inhibitor of eukaryotic translation initiation factor 4E (eIF4E), upon phosphorylation, dissociates from eIF4E, thereby relieving its inhibitory effect on protein synthesis (Wang et al. 2006) (see Fig. 1). This coordinated regulation ensures that mTORC1 promotes anabolic processes necessary for cell growth and proliferation. Sestrin2, CASTOR1, and SAMTOR have been identified as sensors for leucine, arginine, and methionine (Gu et al. 2017; Wolfson et al. 2016; Chantranupong et al. 2016). Glutamine and asparagine have been shown to activate mTORC1, a process that depends on the involvement of ADP-ribosylation factor-1 (Arf1) (Meng et al. 2020; Jewell et al. 2015). A recent study has uncovered a novel amino acid-sensing mechanism for mTORC1. Upon amino acid deprivation, GCN2 is activated, leading to enhanced activity of F-box only protein 22 (FBXO22). FBXO22 mediates the ubiquitination of mTORC1, which disrupts its interaction with substrates and subsequently suppresses mTORC1 activity (Ge et al. 2023).

### Key metabolic pathways

Serine can be acquired by cells through two pathways, one is transported by alanine-serine-cysteine-threonine transporters (ASCT) 1 and 2 from extracellular environment, and the other is de novo serine synthesis pathway (SSP) (see Fig. 1). The intermediate 3-phosphoglycerate (3PG) of glycolysis can be sequentially catalyzed by phosphoglycerate dehydrogenase (PHGDH), phosphoserine aminotransferase 1 (PSAT1), and phosphoserine phosphatase (PSPH) to convert into L-serine. This process is accompanied by the consumption of glutamate and the production of alpha-ketoglutarate (α-KG) (Mattaini et al. 2016). Serine supports one-carbon metabolism, which comprises the folate cycle, the methionine cycle, and the transsulfuration pathway. This metabolic network is essential for the synthesis of nucleotides, S-adenosylmethionine (SAM), NADPH, and glutathione (GSH). Consequently, serine and one-carbon metabolism play crucial roles in maintaining intracellular amino acid levels, providing nucleotides for DNA and RNA synthesis, maintaining redox balance, and supporting SAM-mediated methylation reactions (Mattaini et al. 2016; Zeng et al. 2019).

Glutamine is taken up from the extracellular environment into the plasma by various amino acid transporters, such as SLC1A5 and SLC38A1 (Bhutia et al. 2015) (see Fig. 1). Once inside the cell, glutamine is converted to glutamate by glutaminase (GLS1 or GLS2) (Curthoys and Watford 1995). Conversely, glutamine synthetase (GS) catalyzes the synthesis of glutamine from glutamate. Glutamate can then be converted by either glutamate dehydrogenase (GLUD1/2) or aminotransferases into α-KG, which enters the tricarboxylic acid (TCA) cycle to generate energy (Moreadith and Lehninger 1984). Glutamine serves as an essential source of nitrogen and carbon. Through the action of aminotransferases, the amino group of glutamine is transferred to other α-keto acids, forming the corresponding amino acids while glutamine is metabolized to α-KG. This α-KG can then undergo a series of reactions to produce glucose, lipids, and nucleotides. Additionally, glutamine maintains redox homeostasis by supplying NADPH and GSH. α-KG can be converted to malate, which can subsequently produce pyruvate and NADPH. And glutamine can also combine with glycine and cysteine to produce GSH, a critical intracellular antioxidant (Jin et al. 2023; Altman et al. 2016) (see Fig. 1).

Arginine is primarily transported into cells by amino acid transporters cationic amino acid transporter (CAT-1 and CAT-2) (Closs 1996). Once inside the cell, arginine can be metabolized by either nitric oxide synthase (NOS) or arginase (see Fig. 1). There are three isoforms of NOS: neuronal NOS (nNOS or NOS1), inducible NOS (iNOS or NOS2), and endothelial NOS (eNOS or NOS3). NOS1 and NOS3 are constitutively expressed isoforms, whereas iNOS is induced in response to specific signals such as LPS, IFN-γ, and TNF-α (Martí and Reith 2021). NOS catalyzes the conversion of arginine to NO and citrulline. Arginase exists in two isoforms: arginase I (ARG1) and arginase II (ARG2). Arginase catalyzes the hydrolysis of arginine into ornithine and urea. Ornithine can be metabolized by ornithine decarboxylase (ODC) to produce polyamines, including putrescine, spermine and spermidine. Additionally, ornithine can be converted by ornithine aminotransferase (OAT) into proline. Ornithine can also be converted to citrulline by ornithine carbamoyltransferase (OTC). Aspartate and citrulline are catalyzed by argininosuccinate synthase 1 (ASS1) to form argininosuccinate, which is then cleaved by argininosuccinate lyase (ASL) into arginine and fumarate.

Tryptophan is an essential amino acid that can only be obtained through the diet. Tryptophan is transported into cells via the SLC7A5/SLC3A2 (LAT1) transporter complex. Tryptophan metabolism includes three main pathways: the kynurenine (Kyn), the 5-hydroxytryptamine (5-HT), and the indole pathways (see Fig. 1). Over 95% of tryptophan is metabolized through the kynurenine pathway (Xue et al. 2023a). In the nervous system, tryptophan can be metabolized to 5-hydroxytryptophan (5-HTP) and 5-HT, serving as important neurotransmitters. Dietary tryptophan is absorbed into the bloodstream through the intestinal epithelium. In the gut, a minor portion of tryptophan is converted by gut microbiota into indole and its derivatives. The aryl hydrocarbon receptor (AhR) is a ligand-activated transcription factor that can be activated by Kyn, indole, and indole derivatives. Activated AhR plays a pivotal role in immune regulation.

## Influence of amino acids on myeloid cell function and polarization

### Glutamine

#### Regulation of macrophages

**Fig. 2:**
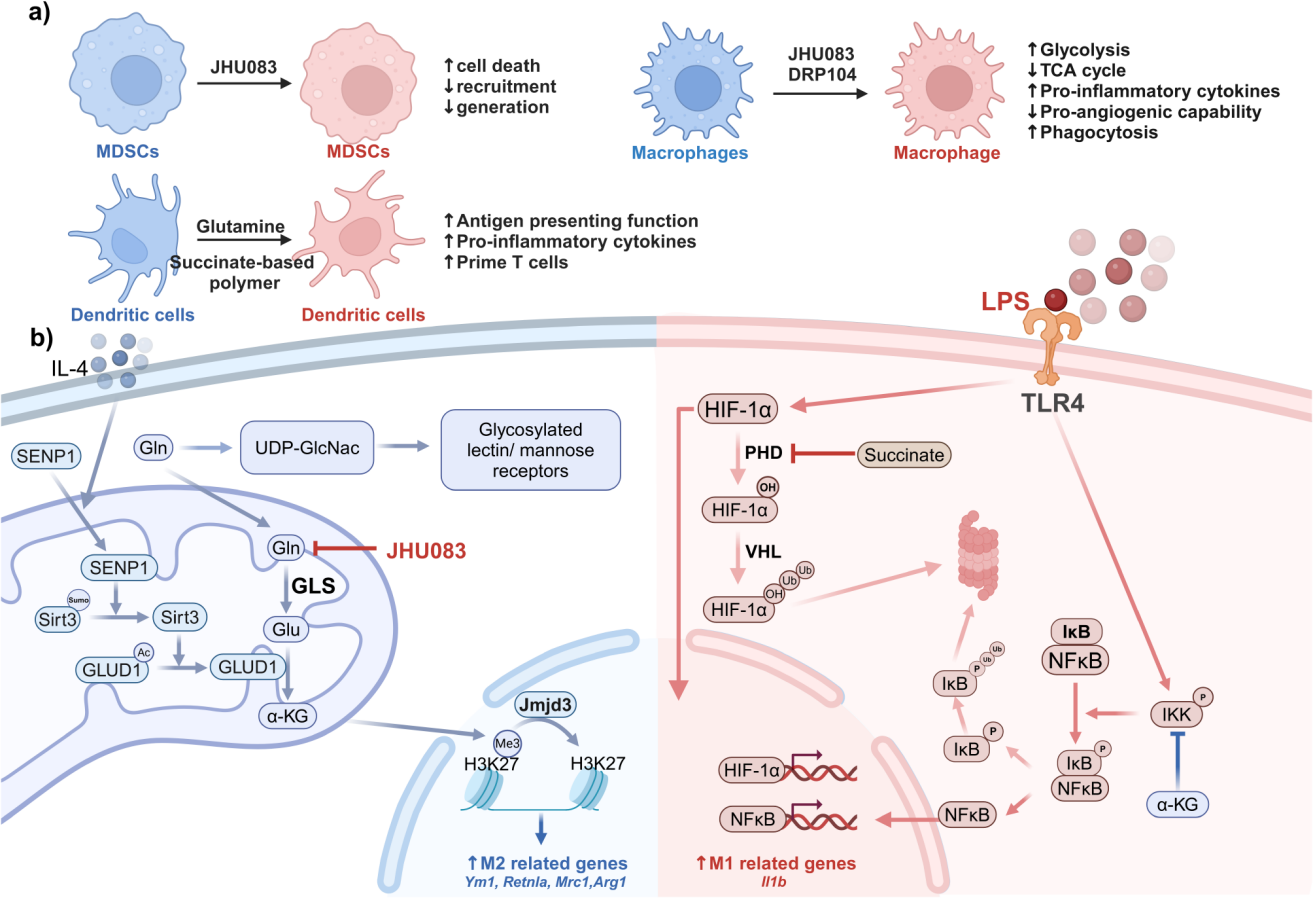
Glutamine associated agents influence myeloid function and glutamine modulates macrophage polarization

Glutamine metabolism plays a pivotal role in macrophage activation, differentiation and functions (see Fig. 2b). Glutamine is processed through the hexosamine biosynthesis pathway to produce UDP-GlcNAc, which is subsequently used in N-glycosylation to generate glycosylated lectin/mannose receptors, prominent markers of M2 polarization. Inhibition of the hexosamine pathway via glucosamine, or inhibition of N-glycosylation reduces the expression of M2 markers, while only minimally affecting M1 polarization (Jha et al. 2015). Furthermore, pharmacological inhibition of glutamine synthetase drives the shift of M2 macrophages towards an M1-like phenotype, characterized by decreased intracellular glutamine, elevated succinate levels and enhanced glycolysis through HIF-1α activation (Palmieri et al. 2017).

Glutamine-derived metabolites, including α-KG, succinate, and aspartate, orchestrate macrophage activation through epigenetic and metabolic reprogramming. Trimethylation of histone H3 at lysine 27 (H3K27me3) suppresses the expression of M2 marker genes such as *Ym1*,* Retnla*,* and Mrc1* (Ishii et al. 2009). α-KG, as an essential cofactor for the demethylase Jumonji domain containing 3 (Jmjd3), promotes H3K27 demethylation, thereby reactivating the expression of M2 marker genes (Liu et al. 2017). Additionally, α-KG inhibits the activation of IKKβ, preventing the nuclear translocation of NF-κB, which reduces the expression of M1-associated inflammatory molecules and impairing M1 polarization (Liu et al. 2017). However, succinate stabilizes hypoxia-inducible factor-1α (HIF-1α), leading to increased expression of IL-1β. Conversely, α-KG can destabilize HIF-1α (Tannahill et al. 2013). Thus, the α-KG/succinate ratio modulates M1/M2 polarization dynamics, with a high ratio promoting M2 polarization and a low ratio favoring M1 polarization (Liu et al. 2017). Furthermore, through the action of glutamate-oxaloacetate transaminase, glutamine is converted to aspartate, which is then used in the synthesis of arginine through the actions of ASS1 and ASL. Arginine, through the polyamine synthesis pathway, is converted into spermidine. Spermidine enhances HIF-1α translation efficiency via eIF5A hypusination. This, in turn, promotes TAM polarization and may drive tumor progression by increasing the secretion of PDGF-A, VEGF-A, and CCL2 (Kim et al. 2024).

Various mechanisms can promote M2 polarization by enhancing the production of α-KG. SUMO-specific protease SENP1 activates Sirtuin 3 (Sirt3), a deacetylase, through de-SUMOylation. Sirt3 then deacetylates glutamate dehydrogenase 1 (GLUD1), thereby increasing GLUD1-catalyzed α-KG production (Zhou et al. 2022). Additionally, gut microbiota-derived glutamine elevates α-KG levels in the blood, promoting M2 polarization and attenuating liver ischemia/reperfusion injury (Lu et al. 2023). This study highlights the potential of modulating gut bacteria as a therapeutic strategy for macrophage reprogramming.

Tumor cells compete with macrophages for glutamine. Glutamine consumption by tumor cells through SLC1A5 transporter disrupts endoplasmic reticulum (ER) homeostasis in myeloid cells, leading to ER stress and the upregulation of GPR109A. This stress response drives MDSCs and TAMs to adopt an immunosuppressive phenotype, facilitating tumor immune evasion (Yang et al. 2024a). Furthermore, glutamine depletion by tumor cells overexpressing glutamine-fructose-6-phosphate transaminase 2 (GFPT2) inhibits mitochondrial fission-mediated cytosolic calcium in macrophages. Reduced calcium levels promote liquid–liquid phase separation, thereby inhibiting macrophage phagocytosis of tumor cells. Supplementing glutamine or targeting GFPT2 can restore macrophage phagocytic activity towards tumor cells (Li et al. 2022).

In cancer treatment, various drugs targeting key enzymes involved in glutamine metabolism can reprogram macrophages, thereby enhancing their antitumor efficacy (see Fig. 2a). The glutamine antagonist DON (6-diazo-5-oxo-L-norleucine) inhibits a range of glutamine-utilizing enzymes, including glutaminase and several glutamine aminotransferases, demonstrating potent antitumor effects (Lemberg et al. 2018). Administration of JHU083, a prodrug of DON, reprograms tumor-associated macrophages (TAMs), resulting in increased glycolysis, disruption of the TCA cycle, and alterations in purine metabolism (Praharaj et al. 2024). This JHU083-induced TAM reprogramming leads to significant tumor growth inhibition in urologic cancers, characterized by enhanced secretion of pro-inflammatory cytokines, increased tumor cell phagocytosis, and reduced pro-angiogenic activity (Praharaj et al. 2024). Similarly, sirpiglenastat (DRP-104), another prodrug of DON, promotes TAM polarization towards the M1 phenotype while simultaneously decreasing PD-L1 expression on TAMs (Yokoyama et al. 2022). Furthermore, the glutamine antagonist synergizes with immune checkpoint blockade therapy (Yokoyama et al. 2022; Oh et al. 2020).

#### Impact on MDSCs

Kumar et al. employed PEGylated *Helicobacter pylori* gamma-glutamyl transferase (PEG-GGT) to efficiently deplete circulating glutamine, resulting in an increased accumulation of MDSCs (Kumar et al. 2024). Subsequent analysis of various human cancers from TCGA database reveal a correlation between glutamine depletion and MDSC accumulation (Kumar et al. 2024). However, pan-inhibition of glutamine-utilizing enzymes reduces MDSC infiltration and enhances anti-tumor immunity. In the ID8 ovarian cancer mouse model, inhibition of glutamine metabolism pathways attenuated α-KG-driven oxidative phosphorylation in MDSCs, leading to reduced expression of immune-suppressive genes and impaired immune inhibitory functions (Udumula et al. 2021). Additionally, in the murine 4T1 breast cancer model, inhibition of glutamine metabolism via JHU083 suppresses the generation and recruitment of MDSCs (Oh et al. 2020). Glutamine inhibition also induces caspase-3–dependent cell death in MDSCs and decreases the CSF3 secretion from tumor cells, which, in turn, reduces MDSC recruitment to the TME. Additionally, glutamine inhibition promotes a proinflammatory macrophage phenotype through enhanced NF-κB activation and reduced STAT3 signaling (Oh et al. 2020). This seemingly contradictory phenomenon arises, in part, from the fact that DON treatment inhibits glutamine metabolism, leading to glutamine accumulation within the tumor microenvironment (TME), whereas PEG-GGT depletes glutamine in the TME but does not inhibit intracellular glutamine metabolism (Kumar et al. 2024). Elucidating the role of different glutamine-utilizing pathways in various cell types within the TME is essential to understand the integrated impact of glutamine metabolism.

Moreover, glutamine enters the TCA cycle to produce itaconate, which downregulates mitochondrial ROS and supports MDSC survival (Daneshmandi et al. 2024).

#### Role in DCs function

Conventional dendritic cells type 1 (cDC1s) are capable of cross-presenting tumor antigens to CD8^+^ cytotoxic T cells and are pivotal in initiating effective anti-tumor responses (Böttcher and Reis e Sousa 2018). However, tumor cells compete with cDC1s for glutamine in the tumor microenvironment via the SLC38A2 transporter, thus impeding cDC1 function and evading T cell-mediated killing (Guo et al. 2023). Intratumoral glutamine supplementation restores the antigen-presenting function of cDC1s and enhances CD8^+^ T cell immunity via the FLCN–TFEB axis (Guo et al. 2023). This study suggests that glutamine functions as a signaling molecule between DCs and tumor cells. Moreover, in response to glutamine deficiency, DCs upregulate GS to synthesize more glutamine, thereby rescuing their survival (Schoeppe et al. 2023). In breast cancer, high expression of GLS and low expression of glutamate-oxaloacetate transaminase 2 (GOT2) correlate positively with the presence of DCs and suggest an enhanced response to immunotherapy (Yang et al. 2024b). However, the precise mechanisms by which GLS and GOT2 modulate DC function warrant further exploration.

Succinate is recognized as an inflammatory signal that induces IL-1β production in macrophages (Tannahill et al. 2013). However, its effect on DCs remains unclear. Inamdar et al. developed a succinate-based polymer that gradually releases succinate, thereby activating DCs through the HIF-1α, TNF-α, and thymic stromal lymphopoietin (TSLP) signaling pathways. This activation enhances the DCs’ ability to produce pro-inflammatory cytokines and prime T cells, leading to a more robust anti-tumor response in a melanoma mouse model (Inamdar et al. 2023).

#### Modulation of Neutrophil Activity

In an ovalbumin (OVA)-based murine asthma model, glutamine deficiency induced by inhibiting ASCT2 and glutamine synthetase disrupts MAPK phosphatase, leading to prolonged MAP kinase activation. This results in increased neutrophil accumulation and elevated release of inflammatory cytokines, thereby exacerbating airway inflammation (Kim et al. 2022).

Glutaminolysis provides energy for neutrophils under conditions of glucose deficiency. In the LPS-induced acute lung injury model, airway neutrophils when exposed to hypoxic and hypoglycemic conditions utilize extracellular glutamine produced during lung injury to sustain their energy supply (Watts et al. 2021). Furthermore, Sadiku et al. demonstrated that glutamine can be utilized for gluconeogenesis, producing intracellular glycogen. This glycogen provides a sustained energy source crucial for the survival and pathogen-killing capabilities of neutrophils (Sadiku et al. 2021). Additionally, glutamine enhances the production of superoxide anions and ROS (Pithon-Curi et al. 2002; Furukawa et al. 2000), which are crucial for neutrophil-mediated pathogen killing. Glutamine also inhibits the neutrophil migration to infection site (Santos et al. 2019).

### Serine

#### Impact on macrophage polarization

**Fig. 3:**
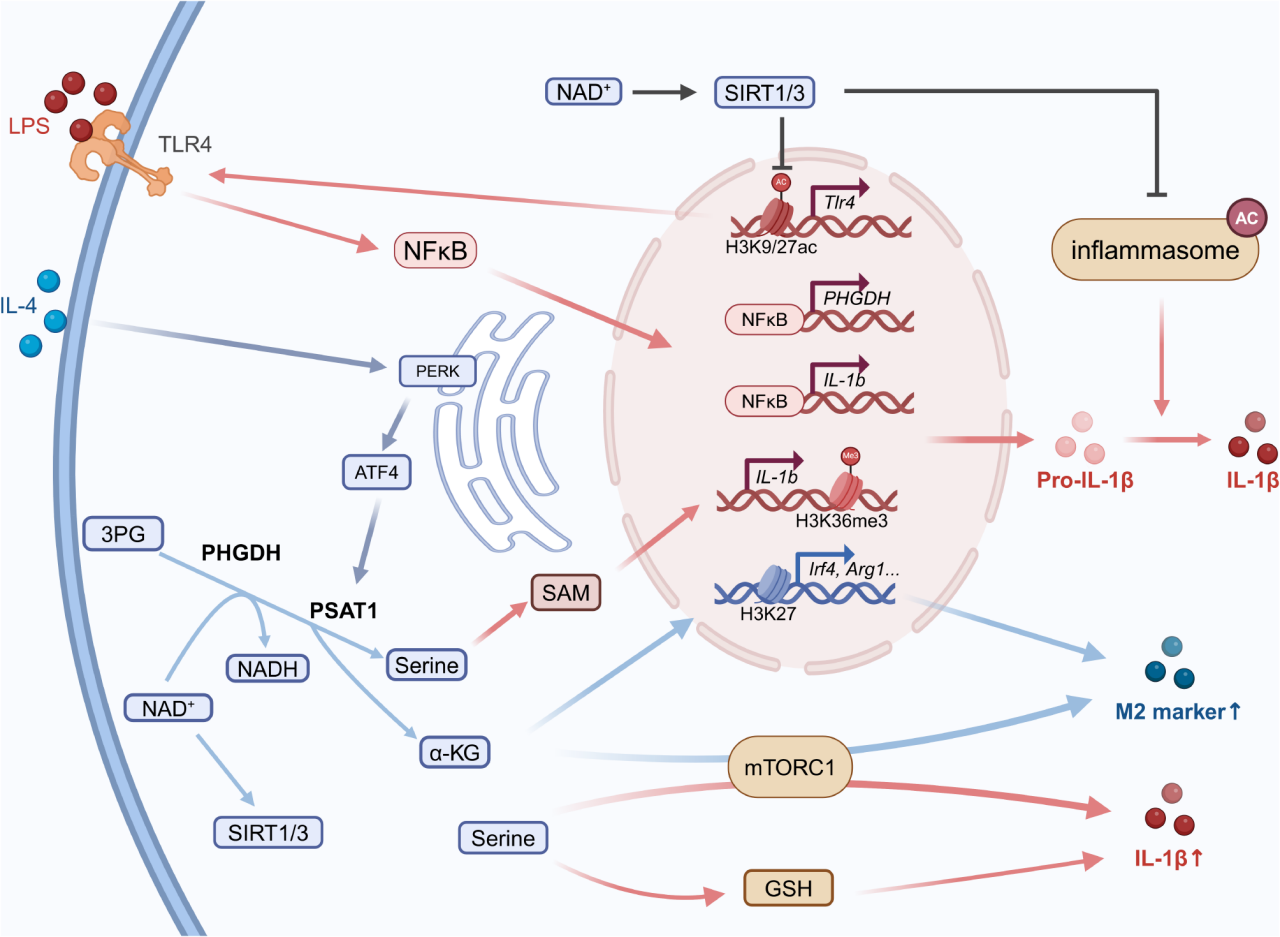
Serine modulates macrophage polarization

Serine metabolism is essential for the production of the pro-inflammatory cytokine IL-1β, operating through several mechanisms (see Fig. 3). These include the generation of GSH (Rodriguez et al. 2019), the mediation of histone H3 lysine 36 trimethylation via SAM (Yu et al. 2019), and the activation of mTOR signaling (Chen et al. 2020). In response to LPS stimulation, the SSP and exogenous serine enhance the production of one-carbon units, supporting de novo ATP synthesis, which subsequently increases SAM production. SAM-dependent H3K36me3 promotes transcription elongation in gene-body regions of certain inflammatory genes, thereby enhancing their expression (Yu et al. 2019). Recent studies have identified PHGDH as a key regulator of IL-1β production through serine-independent mechanisms (Wang et al. 2024). Specifically, PHGDH catalyzes the conversion of 3PG, which simultaneously reduces NAD^+^ to NADH, leading to a decrease in intracellular NAD^+^ levels. This reduction inhibits the activity of NAD^+^-dependent deacetylases SIRT1 and SIRT3, resulting in diminished deacetylation of the H3K9/27 region on the *Tlr4* gene. The subsequent increase in TLR4 expression enhances *Il1b* transcription through the TLR4-NF-κB signaling pathway (Wang et al. 2024). Additionally, reduced SIRT1/3 activity prevents the deacetylation of inflammasome components NLRP3 and ASC at K21/22/24, protecting them from ubiquitination and degradation. This stabilization of inflammasome components promotes the conversion of pro-IL-1β to active IL-1β, thereby maintaining the pro-inflammatory phenotype of macrophages (Wang et al. 2024).

Serine metabolism also plays a pivotal role in the polarization of immunosuppressive M2 macrophages (see Fig. 3). Through inverse data-driven modeling and multi-omics analysis, PHGDH has been identified as a metabolic signature of M2 macrophages, enhancing their polarization (Wilson et al. 2020). Further studies confirm that α-KG produced by the SSP activates mTORC1, a key mediator of M2 polarization (Cai et al. 2024). Elevated PSAT1 expression has been linked to increased transamination and subsequent α-KG production, which facilitates Jmjd3-dependent histone demethylation, thereby promoting the expression of immunosuppressive genes (Raines et al. 2022). Moreover, increased serine levels resulting from elevated PSAT1 expression enhance mitochondrial fitness (Raines et al. 2022). PHGDH also drives the serine-derived synthesis of SAM, which inhibits *Igf1* gene expression through H3K27me3. This inhibition modulates the IGF1-p38-JAK-STAT1/STAT6 axis, suppressing STAT1-mediated M1 polarization while promoting STAT6-mediated M2 polarization (Shan et al. 2022).

Serine metabolism has been implicated in mediating the shift towards an immunosuppressive phenotype in macrophages during chronic inflammation, a process crucial for resolving inflammation. IL-4 stimulates the protein kinase RNA-like ER kinase (PERK), a key sensor of endoplasmic reticulum (ER) stress. PERK activation leads to the activation of ATF4, which, in turn increases PSAT1 expression and enhances serine biosynthesis. The elevated levels of α -KG and serine, products of this metabolic pathway, further drive the polarization of macrophages towards an immunosuppressive subtype, facilitating the resolution of inflammation (Raines et al. 2022).

The CCL2-CCR2 chemokine signaling pathway is critical for recruiting monocytes/macrophages from the vasculature to the tumor site (Anderson et al. 2021), a key step in the formation of tumor-associated macrophages (TAMs) that shape the tumor immune landscape. PHGDH deficiency reduces CCR2 expression in TAMs, likely through the mTORC1 signaling pathway, which impairs macrophage migration toward tumor cells (Cai et al. 2024). In murine models, macrophage-specific genetic ablation of PHGDH diminishes the infiltration of immunosuppressive TAMs and induces a phenotypic shift from M2-like to M1-like TAMs. This shift enhances antitumor T-cell immunity and slows tumor progression (Cai et al. 2024).

Interestingly, serine derived from tumor cells can also influence macrophage polarization. Tumor cells with FGFR3 mutations upregulate PSAT1, enhancing serine synthesis. This newly synthesized serine can be exported to macrophages, where it methylates PI3K and activates the PI3K/Akt pathway, promoting M2 macrophage polarization (Ouyang et al. 2023). This shift impairs macrophages’ ability to recruit T cells and present antigens, thereby facilitating tumor progression (Ouyang et al. 2023). Consequently, this mechanism contributes to the resistance of FGFR3-mutant bladder cancer to the FGFR tyrosine kinase inhibitor erdafitinib. The PI3K-targeted inhibitor duvelisib can reverse macrophage polarization toward the M2 phenotype and restore their immunostimulatory functions. Therefore, the combination of duvelisib and erdafitinib exhibits enhanced antitumor activity (Ouyang et al. 2023).

The paradoxical effects of serine on macrophage polarization may stem from differences in polarization time and the experimental model used. Depletion of serine/glycine or inhibition of serine transporters significantly reduced *Il-1β* mRNA levels in LPS-stimulated BMDMs at 4 h (Rodriguez et al. 2019), but increased both the mRNA and protein levels of IL-1β in BMDMs after 12 h of IFN-γ stimulation or 24 h of LPS stimulation (Wilson et al. 2020; Shan et al. 2022). These findings suggest that the effects of serine on macrophage polarization are highly context-dependent. Further studies should investigate the impact of serine on macrophages in various polarization states. In addition, the various signals derived from serine are intricately intertwined. The use of isotopic labeling of serine or glycine could help clarify under which conditions serine is converted into certain substances, thereby influencing the polarization direction of macrophages.

#### Role in DCs function

Recent studies have highlighted the pivotal role of serine in regulating plasmacytoid dendritic cell (pDC) function in autoimmune diseases. The activation of ER stress triggers the IRE1α-XBP1–PHGDH axis, leading to enhanced serine biosynthesis. This upregulation reroutes the glycolytic intermediate 3-PG away from its typical role in glycolysis, thereby reducing the flux of pyruvate into the TCA cycle. This metabolic reprogramming results in diminished ATP production and subsequently attenuates type I interferon (IFN-I) responses in pDCs (Chaudhary et al. 2022). Thus, serine metabolism emerges as a crucial modulator, potentially mitigating the severity of autoimmune diseases.

#### Modulation of Neutrophil Activity

In liver cancer cells, nuclear PHGDH interacts with nuclear c-Myc to form a complex, which drives the expression of CXCL1 and IL-8 via the PHGDH/p300/c-Myc/AF9 transactivation axis. This mechanism recruits neutrophils and TAMs to the liver, thereby accelerating the progression of hepatocellular carcinoma (Zhu et al. 2023). This study reveals that the intracellular localization of metabolic enzymes influences their functional roles. Notably, cytosolic PHGDH serves as a key enzyme in the SSP, while nuclear PHGDH plays a non-metabolic role.

### Arginine

#### Influence on macrophage function

Arginine metabolism, particularly the equilibrium between NO production and arginase activity, profoundly influences macrophage polarization and function. M1 macrophages are characterized by elevated iNOS activity, leading to increased NO production, which drives pro-inflammatory responses and host defense against pathogens. Conversely, M2 macrophages demonstrate heightened arginase activity, leading to increased ornithine production. This supports tissue repair, immunoregulation, and resolution of inflammation. Consequently, iNOS and Arg1 expression serve as hallmarks of M1 and M2 macrophages, respectively (Mills et al. 2000). iNOS expression is synergistically induced by STAT1, downstream of IFN-γ receptor signaling, and NF-kB, downstream of LPS Toll-like receptor (TLR) signaling. Conversely, Arg1 expression is induced by STAT6, downstream of IL-4 receptor signaling (Ji et al. 2019).

**Fig. 4:**
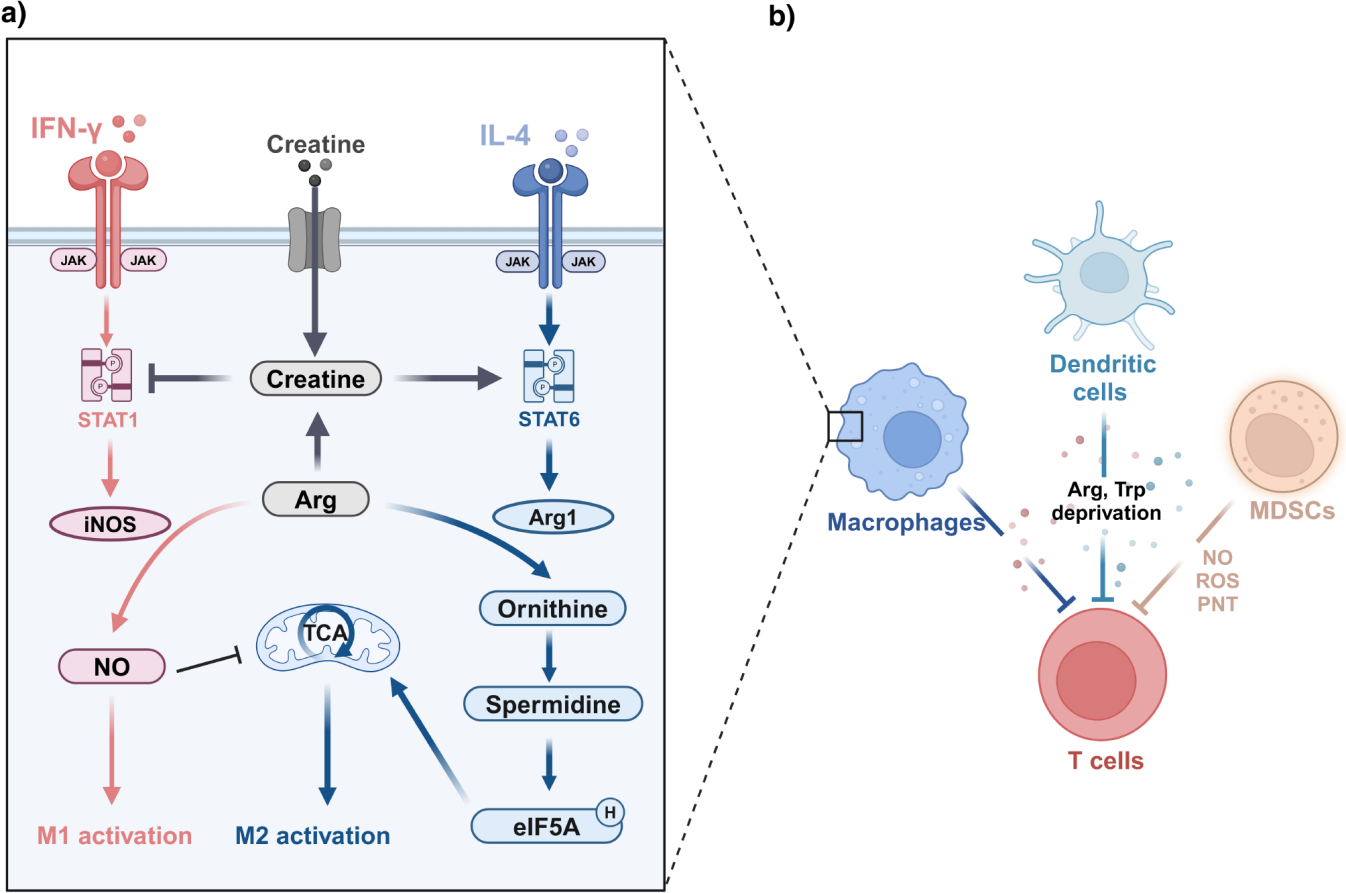
Arginine modulates macrophage polarization and mediates the inhibitory effect of myeloid cells on T cells

Arginine metabolism plays a crucial role in the metabolic reprogramming of macrophages (see Fig. 4). NO disrupts the TCA cycle by targeting aconitase 2 and pyruvate dehydrogenase, rerouting macrophages into glycolysis. This metabolic shift promotes the polarization of macrophages towards the M1 phenotype (Palmieri et al. 2020, 2023). Conversely, polyamines derived from ornithine, such as spermidine, facilitates the hypusination of eIF5A (eIF5A^H^), thereby promoting the expression of a specific subset of mitochondrial proteins that are integral to the TCA cycle and OXPHOS. Additionally, eIF5A^H^ contributes to the efficient translation of mitochondrial targeting sequences (MTSs) in certain proteins, which guide these proteins into the mitochondria (Puleston et al. 2019). Consequently, polyamines induce metabolic reprogramming in macrophages through epigenetic modifications, thereby promoting M2 polarization.

Other byproducts of arginine metabolism also impact the functionality of macrophages. Arginine can be metabolized to produce creatine, which can also be imported by macrophages via the SLC6A8 transporter. Creatine inhibits the phosphorylation of STAT1, suppressing iNOS expression. Simultaneously, creatine enhances STAT6 phosphorylation, promotes chromatin remodeling, and upregulates the expression of Arg1 (Ji et al. 2019). These processes collectively facilitate the polarization of macrophages towards an M2-like phenotype. Moreover, TAMs supply creatine to tumors, helping them to withstand hypoxia-induced stress and facilitating glioblastoma progression (Rashidi et al. 2024). In glioblastoma, TAMs produce large amounts of polyamines to buffer the low intracellular pH, enabling their survival in the harsh acidic environment of solid tumors. Furthermore, blocking polyamine synthesis can significantly improve survival rates in glioblastoma models (Miska et al. 2021).

Sepiapterin, an endogenously-produced precursor of the NOS cofactor tetrahydrobiopterin (BH4), can shift arginine metabolism from the polyamine synthesis pathway to the NO synthesis pathway in breast cancer. This shift mediates the transition of M2 TAMs to M1 TAMs (Zheng et al. 2020). Additionally, sepiapterin can inhibit PD-L1 expression in tumor cells, thereby slowing tumor progression (Zheng et al. 2020).

A range of drugs have been shown to successfully reprogram TAM phenotypes from M2 to M1, including anti-CD47 and anti-CD40 antibodies, CSF1R and PI3Kγ inhibitors, and Toll-like receptor agonists (Zheng et al. 2024). However, inhibitory cytokines and metabolic byproducts present in the tumor microenvironment (TME), such as IL-4, IL-13, TGF-β, and lactate, can induce the reversion of polarized M1 macrophages back to the M2 phenotype. This negative feedback mechanism attenuates the efficacy of TAM-polarizing agents (Zheng et al. 2024). To address this challenge, Zheng et al. developed an arginine nano-assembly loaded with the Toll-like receptor 7/8 agonist resiquimod (R848) (R848@Arg). Resiquimod repolarizes macrophages, while arginine is metabolized by iNOS to produce NO. NO disrupts mitochondrial function by inhibiting OXPHOS, thereby preventing the transition from M1 to M2 macrophages, maintaining M1 macrophage infiltration in tumors, and inhibiting tumor progression (Zheng et al. 2024).

#### Role in MDSC-Mediated immunosuppression

In the TME, MDSCs rely on iNOS and Arg1 to deplete arginine, thereby exerting their immunosuppressive functions. Upon recruitment to the tumor site, MDSCs upregulate the expression of CAT2, enhancing arginine uptake. The loss of CAT2 diminishes the immunosuppressive capabilities of MDSCs, resulting in increased T cell proliferation and enhanced antitumor activity (Cimen Bozkus et al. 2015). MDSCs also upregulate Arg1 to further deplete arginine, which decreases the expression of the T cell receptor CD3ζ chain and impairs T cell responses (Rodriguez et al. 2004). Additionally, MDSCs suppress T cell function through NO-dependent pathways. NO can directly induce T cell apoptosis by impairing the IL-2R signaling pathway, including the inhibition of Janus kinase (JAK)1 and JAK3, STAT5, extracellular signal-regulated kinase (Erk), and Akt phosphorylation (Mazzoni et al. 2002). Moreover, NO can induce DNA damage and activate the p53 pathway in CD8 ^+^ T cells, inducing T cell apoptosis (Cartwright et al. 2021). In conditions of low extracellular arginine, iNOS mediates the production of superoxide (O_2_^−^), which reacts with other molecules to generate peroxynitrite (PNT) and reactive oxygen species (ROS) (Bronte and Zanovello 2005). PNT can render cytotoxic T lymphocytes unresponsive, thereby promoting tumor progression (Bronte et al. 2005). Additionally, ROS and PNT produced by MDSCs can induce tyrosine nitration of the TCR-CD8 complex, disrupting the binding of peptide-major histocompatibility complex (pMHC) dimers to CD8-expressing T cells, thereby impairing T cell activation and their antitumor functions (Nagaraj et al. 2007).

PMN-MDSCs predominantly employ ROS, PNT, and arginase 1 to exert their immunosuppressive functions. In contrast, M-MDSCs are more inclined to produce NO and release immunosuppressive cytokines such as IL-10 and TGF-β (Veglia et al. 2021; Gabrilovich 2017).

A recent study demonstrates that the V-domain suppressor of T cell activation activates STAT3 and upregulates the expression of ARG1, increasing the production of polyamines. Consequently, this drives the differentiation of MDSCs (Zhang et al. 2024).

#### Regulation of dendritic cells

Arginine metabolism is also considered a characteristic feature of suppressive DCs. NO produced by DC inhibits mitochondrial activity and OXPHOS, driving DCs to rely on glycolysis for energy production, thereby impairing their function (Thwe and Amiel 2018). Inhibition of NO production enhances DC activation, increasing their ability to stimulate T cells and promote T cell survival (Thwe and Amiel 2018). Nω-nitro-L-arginine methyl ester (L-NAME) and N-hydroxy-nor-L-arginine (nor-NOHA) are inhibitors of iNOS and arginase, respectively. Treatment with L-NAME increased CD86 expression in bone marrow-derived dendritic cells (BMDCs), whereas treatment with NOHA increased both CD80 and CD86 expression, both of which are co-stimulatory molecules (Simioni et al. 2017). This study suggests that modulation of dendritic cell arginine metabolism could potentially serve as a target for regulating immune response.

TNF-α and iNOS-producing dendritic cells (Tip-DCs) represent a specific subpopulation of dendritic cells that express TNF-α and NOS2. Tip-DCs exert pro-inflammatory effects during bacterial infections (Serbina et al. 2003). Furthermore, NO derived from Tip-DCs enhances the tumor-killing function of adoptively transferred CD8^+^ cytotoxic T cells (Marigo et al. 2016), providing new evidence for the anti-tumor role of NO.

Tumor-infiltrating dendritic cells (TIDCs) decrease the expression of CD3ζ in T cells through the action of ARG1, inducing anergy in CD8^+^ T cells and thereby promoting tumor progression (Norian et al. 2009).

#### Effects on neutrophils

Human neutrophils constitutively express high levels of arginase 1, an enzyme that can also be released into the extracellular space, depleting extracellular arginine and suppressing T-cell functions (Munder et al. 2006). Interestingly, arginase 1 released by activated neutrophils or from dead cells can induce apoptosis in cancers by triggering ER stress. The arginase inhibitor NOHA attenuates both apoptosis and the ER stress response (García-Navas et al. 2021). The dual role of arginase in anti-tumor responses warrants further investigation.

Inflammatory stimuli can upregulate the expression of MET, a tyrosine kinase receptor. Upon binding with hepatocyte growth factor (HGF), MET induces neutrophil transmigration across activated endothelium and increases iNOS expression. The HGF/MET-dependent NO promotes cancer cell killing, thereby reducing tumor growth and metastasis (Finisguerra et al. 2015).

### Tryptophan

#### Role in macrophage polarization

Tryptophan metabolism influences macrophage polarization. In THP-1 cells, overexpression of IDO promotes macrophage polarization toward the M2 phenotype, whereas IDO knockout shifts macrophages toward the M1 phenotype (Wang et al. 2014). Furthermore, IDO1 activates GCN2, leading to increased expression of IL-10 and decreased expression of IL-12 in macrophages, thereby supporting immune tolerance (Ravishankar et al. 2015). In breast cancer, Xue et al. identified a positive correlation between macrophage tryptophan metabolic activity and M1 polarization through transcriptomic analysis. In vitro experiments further confirmed that tryptophan metabolism can promote macrophage M1 polarization. This research highlights the predictive value of macrophage tryptophan metabolism in assessing the efficacy of breast cancer immunotherapy (Xue et al. 2023b). In addition, *Lactobacilli* can metabolize dietary tryptophan into indoles, which activate AhR in macrophages, driving TAMs to adopt an immunosuppressive phenotype. Depletion of dietary tryptophan reduces AhR activity in TAMs, promoting the accumulation of TNF-α^+^IFN-γ^+^CD8^+^ T cells within the tumor and slowing tumor progression (Hezaveh et al. 2022). This finding underscores the significant impact of microbial amino acid metabolism on myeloid cell function.

Tryptophan metabolism of tumor cells also affect macrophage function. In B16-F10 melanoma models overexpressing IDO or TDO (B16IDO or B16TDO), B16IDO or B16TDO cells metabolize tryptophan into Kyn, activating the Kyn-AhR pathway, which suppresses the antigen-presenting function of macrophages and their ability to prime T cells (Campesato et al. 2020). Additionally, B16IDO promotes the polarization of macrophages towards the M2 phenotype (Campesato et al. 2020). In B16IDO tumors, TAMs also interact with Tregs, enhancing immunosuppression. Blocking AhR delays the progression of IDO/TDO-overexpressing tumors and increases the sensitivity of cancer cells to anti-PD-1 therapy (Campesato et al. 2020).Glioblastoma-derived Kyn activates the AhR in TAMs, promoting several mechanisms that drive tumor progression. First, Kyn-activated AhR upregulates the expression of CCR2, facilitating the recruitment of TAMs to GBM. Additionally, Kyn enhances the expression of KLF4, which inhibits NF-κB activation, thereby polarizing TAMs towards the M2 phenotype. Furthermore, Kyn promotes the expression of the ectonucleotidase CD39, which, in cooperation with CD73, generates adenosine. This process impairs the function of CD8 + T cells, further contributing to tumor immune evasion (Takenaka et al. 2019).

#### Modulation of MDSC function

MDSCs can induce the expression of IDO, which subsequently reduces tryptophan levels in the microenvironment. In breast cancer, STAT3-dependent upregulation of IDO mediates the suppression of T cell proliferation and Th1 polarization, while promoting T cell apoptosis and the release of immunosuppressive cytokines (Yu et al. 2013). Depletion of tryptophan in the microenvironment leads to downregulation of T cell TCR-CD3 and inhibition of mTORC1, inducing T cell anergy (Grohmann and Bronte 2010; Lee et al. 2002). IDO1 activates GCN2 by depleting tryptophan, which in turn induces a subset of MDSCs to release IL-6, thereby promoting angiogenesis (Dey et al. 2021).

#### Influence on DCs

Tryptophan metabolism plays a pivotal role in mediating the immunosuppressive functions of DCs. In mouse tumor-draining lymph nodes (LN), researchers have identified a small population (approximately 0.5% of LN cells) of pDCs that highly express IDO. In vitro experiments have demonstrated that these IDO-expressing pDCs can potently inhibit T cell responses, not only to antigens presented by themselves but also to those presented by other antigen-presenting cells (APCs) (Munn et al. 2004).

Among the metabolites of tryptophan, 3-hydroxyanthranilic acid (3-HAA) has been shown to decrease the production of pro-inflammatory cytokines, such as IL-12, IL-6, and TNF-α and reduce the expression of maturation markers (CD40, CD80, and CD86) in LPS-stimulated BMDCs, thereby affecting DC activation. Furthermore, 3-HAA attenuates the ability of DCs to promote T cell activation and differentiation (Lee et al. 2013). Additionally, indole, a product of gut bacteria, can be metabolized in the liver to form indoxyl 3-sulfate (I3S), which inhibits the maturation and activation of monocyte-derived dendritic cells (Ghimire et al. 2018). The AhR has been found to increase the expression of IDO1 in DCs, thereby promoting the release of Kyn and inducing the differentiation of naïve T cells into Treg cells, contributing to immunosuppression (Nguyen et al. 2010).

In addition to tryptophan metabolism, crosstalk in amino acid metabolism, both within and between cells, underpins the immunosuppressive functions of dendritic cells. Under homeostatic conditions, only mature CCR7^+^ cDC1 cells express IDO1, a process dependent on interferon regulatory factor 8 (IRF8). cDC2 cells and pDCs do not express IDO1 (Gargaro et al. 2022). However, IDO1-expressing cDC1 cells can induce IDO1 expression in cDC2 cells through the production of Kyn, revealing a metabolic communication pathway that helps maintain self-tolerance (Gargaro et al. 2022). TGF-β promotes the upregulation of Arg1 and IDO1 expression in DCs, leading to the depletion of extracellular amino acids and contributing to their immunosuppressive function. Moreover, spermidine produced by upregulated Arg1 activates Src kinase, which phosphorylates the immune-based inhibitory tyrosine motifs (ITIMs) within the intracellular domain of IDO1. This phosphorylation cascade initiates signaling pathways that enable DCs to exert long-term immunosuppressive effects (Mondanelli et al. 2017). Similarly, spermidine from bystander Arg1^+^ MDSCs exerts comparable effects (Mondanelli et al. 2017).

### Other amino acids

#### Cysteine

Cells acquire cysteine through two primary pathways. The first involves plasma membrane cystine transporters, which import disulfide-bonded cystine into the cell, where it is subsequently reduced to cysteine. The second pathway is intracellular synthesis via cystathionase, which converts methionine into cysteine (See Fig. 1). Cysteine is crucial for T cell activation. However, T cells lack both cystathionase and cystine transporters, making them dependent on macrophages and/or dendritic cells to supply cysteine. MDSCs also lack the ability to synthesize cysteine and must rely on the import of extracellular cystine, which is reduced to cysteine. This mechanism enables MDSCs to inhibit T cell access to cysteine, thereby exerting an immunosuppressive effect (Srivastava et al. 2010).

#### Methionine

When macrophages engulf apoptotic cells, they utilize methionine derived from these cells to synthesize SAM. SAM subsequently methylates the extracellular signal-regulated kinase 1/2 (ERK1/2) phosphatase Dusp4, which diminishes the inhibitory effect of Dusp4 on phosphorylated ERK1/2. This process enhances prostaglandin E2 (PGE2) synthesis and induces Ptgs2 expression, ultimately promoting tissue resolution (Ampomah et al. 2022).

#### Phenylalanine

Phenylalanine activates the calcium-sensing receptor (CaSR), initiating the NLRP3 pathway, which promotes pyroptosis in macrophages and exacerbates inflammation (Tang et al. 2023). Additionally, another study demonstrated that phenylalanine upregulates the expression of amino acid transporters LAT1 and LAT2 in macrophages, thereby increasing valine uptake. The succinyl-CoA generated from valine metabolism can enter the TCA cycle, enhancing OXPHOS and reducing the release of inflammatory molecules from macrophages (Zhang et al. 2023a). This dual role of phenylalanine may result from variations in concentration and treatment conditions across the in vitro experiments of the two studies; the first study employed a phenylalanine concentration of 600 µM for 4 h, while the second utilized concentrations of 1 or 10 mM for durations of 12–24 h. Further investigation is needed to elucidate the effects of phenylalanine concentration and treatment duration on macrophages.

#### Branched chain amino acids

Branched chain amino acids (BCAAs) including leucine, isoleucine and valine, play a crucial role in cellular metabolism by serving as a major carbon source, facilitating glutamine production, and acting as substrates for the generation of acetyl-CoA and succinyl-CoA, essential components of the TCA cycle (Neinast et al. 2019).

The transporter SLC7A5 and SLC3A2 facilitates the uptake of BCAAs and aromatic amino acids into cells. In human monocytes and macrophages, SLC7A5-mediated amino acids influx activates mTORC1, leading to increased production of IL-1β and TNF-α (Yoon et al. 2018). Additionally, researchers discovered that the inhibition of SLC7A5-mediated amino acid influx significantly diminishes glycolytic activity in human macrophages, as indicated by a reduction in the extracellular acidification rate (ECAR) (Yoon et al. 2018). This finding highlights the interplay between amino acid metabolism and glycolysis, although the underlying mechanisms warrant further investigation.

Leucine can be metabolized into acetyl-CoA, which mediates histone H3K27ac modifications, thereby upregulating HLA-DR expression in neutrophils and enhancing their antigen-presenting capability. This process initiates an antigen-specific T-cell response. In murine models, a leucine-enriched diet enhanced the efficacy of anti-PD-1 therapy (Wu et al. 2024).

Furthermore, leucine promotes the differentiation of common myeloid progenitors (CMP) into polymorphonuclear PMN-MDSCs in the bone marrow by activating the mTORC1 signaling pathway (Chen et al. 2024). This process increases the levels of PMN-MDSCs in both circulating blood and the tumor microenvironment, thereby facilitating tumor progression. Notably, elevated levels of leucine result from the metabolism of gut microbiota associated with a high-fat diet (Chen et al. 2024).

## Amino acid sensing mechanisms

### Role of GCN2 in myeloid cell function

#### Impact on macrophage polarization

GCN2 regulates macrophage polarization and phagocytic function. It can be activated by IDO1, leading to increased IL-10 expression and decreased IL-12 expression in macrophages. This balance supports the maintenance of immune tolerance induced by apoptotic cells. Furthermore, GCN2 agonists have been shown to alleviate autoimmunity in murine models (Ravishankar et al. 2015). In diabetic mouse models, GCN2 inhibits M1 macrophages polarization through the OXPHOS-ROS-NF-κB pathway, while promoting M2 macrophage polarization via the eIF2α-HIF-1α-glycolysis pathway. This dual regulation facilitates wound healing (Hou et al. 2024). However, in a mouse model of septicemia, tryptophan depletion activates GCN2, which, through the ATF4 and CHOP/GADD153 pathways, enhances macrophage sensitivity to LPS stimulation, resulting in increased IL-6 release (Liu et al. 2014).

Halaby et al. demonstrated that GCN2 influences the function of TAMs and MDSCs in the TME through the CREB-2/ATF4 signaling pathway. Specifically, myeloid-lineage deletion of GCN2 causes TAMs to adopt a pro-inflammatory phenotype, characterized by increased IL-1β release. This knockout also diminishes the suppressive effects of TAMs and MDSCs on intratumoral CD8^+^ T cells, leading to enhanced interferon-γ expression. Consequently, these changes collectively contribute to slowed tumor progression (Halaby et al. 2019).

GCN2 also regulates lysosome maturation and erythrophagocytosis in hepatic macrophages, thereby influencing red blood cell clearance and iron recycling (Toboz et al. 2022).

#### Modulation of MDSC function

GCN2 regulates various functions of MDSCs, including their proliferation and differentiation, inhibition of T cell function, and promotion of angiogenesis. As is previously discussed, GCN2 enhances the immunosuppressive functions of MDSCs (Halaby et al. 2019). Additionally, compound 39, a GCN2 inhibitor, has been shown to alleviate MDSC-mediated T cell suppression and restore T cell proliferation. In the LL2 syngeneic mouse model, treatment with compound 39 significantly delayed tumor progression (Jackson et al. 2022). The BRAF inhibitor dabrafenib can dampen the ability of MDSCs to suppress T cells, exhibiting beneficial off-target effects. Mechanistically, dabrafenib activates GCN2, enhances oxidative respiration, and attenuates the transcriptional programs required for PMN-MDSCs development. This leads to a block in the transition from monocytic progenitors to PMN-MDSCs and induces developmental arrest in PMN-MDSCs (Ciudad et al. 2024). Additionally, upon sensing tryptophan deprivation mediated by IDO1, GCN2 induces a subset of MDSCs to release IL-6, thereby promoting angiogenesis (Dey et al. 2021).

#### Role in DCs function

GCN2, activated by tryptophan deprivation, promotes the formation of tolerogenic DCs, thereby inducing the development of CD4^+^CD25^+^ Foxp3^+^ regulatory T cells (Brenk et al. 2009). The yellow fever vaccine YF-17D activates GCN2, which further enhances the antigen-presenting function of DCs to both CD4^+^ and CD8^+^ T cells (Ravindran et al. 2014).

### Role of mTORC1 in myeloid cell function

#### Impact on macrophage polarization

The tuberous sclerosis complex (TSC), comprising TSC1 and TSC2, inhibits the activity of mTORC1. Myeloid-specific deletion of Tsc1 results in constitutive mTORC1 activation, promoting M1 macrophage polarization characterized by enhanced production of proinflammatory cytokines and reduced secretion of IL-10 (Byles et al. 2013). Moreover, serine promotes the production of IL-1β in macrophages by activating mTORC1(Chen et al. 2020). Conversely, α-KG activates mTORC1 signaling, contributing to the M2 macrophage polarization (Cai et al. 2024).

mTORC1 also regulates the competitive interaction between TAMs and tumor cells. In a breast cancer model, a low-protein diet activates the mTORC1 pathway in macrophages via TFEB and TFE3-dependent mechanisms, while inhibiting mTORC1 signaling in tumor cells, thereby slowing tumor growth (Zhang et al. 2023b).

#### Modulation of MDSC function

The gut microbiota associated with obesity releases significant amounts of leucine, which activates mTORC1 signaling. This activation promotes the differentiation of PMN-MDSCs, thereby facilitating cancer progression (Chen et al. 2024). In ovarian cancer, the dual mTORC1/2 inhibitor AZD2014 effectively diminishes the accumulation of MDSCs and inhibits tumor growth (Pi et al. 2021).

In addition to inhibiting T cell function and promoting tumor progression, MDSCs can also mitigate acute kidney injury by inducing immune tolerance. mTOR deficiency enhances the immunosuppressive function of MDSCs by recruiting and inducing them, thereby improving their protective role (Zhang et al. 2017). Another study confirmed that mTOR deficiency also enhances the protective role of MDSCs in reducing heart transplant rejection (Li et al. 2021).

#### Role in DCs function

mTOR orchestrates diverse functions in DCs, including cytokine production, autophagy, lysosome function, and antigen presentation (Sukhbaatar et al. 2016; Snyder and Amiel 2018). A recent research has revealed that mTORC1 in pDCs promotes the production of type I interferon, tumor necrosis factor, and chemokines, as well as the expression of the cystine transporter SLC7A11 in response to amino acid levels (Grzes et al. 2021).

## Targeting metabolism-related pathways in clinical trials

Several agents targeting amino acid metabolism pathways have been developed to enhance antitumor effects, driven by the impact of amino acid metabolism on myeloid cells. Table 1 summarizes selective clinical trials that report their efficacy and the incidence of adverse events.

**Table 1:** Selected clinical trials involving drugs targeting key enzymes in amino acid metabolism

Drug	Combination partner	Disease	Treatment strategy	Efficacy	Adverse events (AE)	Trial phase and trial identifier	Reference
GLS inhibitors							
Telaglenastat (CB-839)	None	Advanced and/or treatment-refractory solid tumors	Telaglenastat	DCR: 43%	Fatigue (23%) and nausea (19%)	1(NCT02071862)	(Harding et al. 2021)
	Everolimus	Advanced/metastatic RCC	Telaglenastat plus everolimus vs. placebo plus everolimus	mPFS: 3.8 vs. 1.9 months;	Grade ≥ 3 AE: 74% vs. 61%	2(NCT03163667)	(Lee et al. 2022)
	Cabozantinib	RCC	Telaglenastat plus cabozantinib vs. placebo plus cabozantinib	mPFS: 9.2 vs. 9.3 months	Grade ≥ 3 TEAEs: 71% vs. 79%	2(NCT03428217)	(Tannir et al. 2022)
ARG inhibitors							
INCB001158	Gemcitabine Cisplatin	BTC	INCB001158 plus gemcitabine plus cisplatin	ORR: 24.2%; mPFS: 8.5 months	AE: 88%; AEs related specifically to INCB001158: 73%	2 (NCT03314935)	(Javle et al. 2021)
	Pembrolizumab	Microsatellite-stable CRC	INCB001158 plus pembrolizumab	DCR: 30%	None	2(NCT02903914)	(Naing et al. 2019)
iNOS inhibitors							
L-NMMA	Docetaxel	Metastatic Triple Negative Breast Cancer	L-NMMA plus docetaxel	ORR: 55%	SAEs: 15%	1|2(NCT02834403)	None
IDO1 inhibitors							
Epacadostat (INCB024360)	Pembrolizumab	Advanced solid tumors	Epacadostat plus pembrolizumab	ORR: 55%	TRAEs: 84%, Grade ≥ 3 TRAEs: 24%	1/2(NCT02178722)	(Mitchell et al. 2018)
	Pembrolizumab	Melanoma	Epacadostat plus pembrolizumab vs. placebo plus pembrolizumab	ORR: 34.2% vs. 31.5%; PFS: median 4.7 vs. 4.9 months	TRAEs: 79% vs. 81%; Grade ≥ 3 TRAEs: 22% vs.17%	3(NCT02752074)	(Long et al. 2019)
	Pembrolizumab	UC	Epacadostat plus pembrolizumab vs. placebo plus pembrolizumab	ORR: 31.8% vs. 24.5%	TRAEs: 58.1% vs. 59.2%; Grade ≥ 3 TRAEs: 20.9% vs. 14.3%	3(NCT03361865)	(Necchi et al. 2024)
	Pembrolizumab	UC	Epacadostat plus pembrolizumab vs. placebo plus pembrolizumab	ORR: 26.2% vs. 11.9%	TRAEs: 57.1% vs. 53.7%; Grade ≥ 3 TRAEs: 16.7% vs. 7.3%	3(NCT03374488)	(Cicin et al. 2024)
	Durvalumab	Multiple advanced solid tumors	Epacadostat plus durvalumab	ORR: 12.0%	TRAEs: 80.3%; Grade ≥ 3 TRAEs: 18.3%	1/2(NCT02318277)	(Naing et al. 2023)
	Pembrolizumab Chemotherapy	Advanced or metastatic solid tumors	Epacadostat plus pembrolizumab plus chemotherapy	ORR: 31.4%	Grade ≥ 3 TEAEs: 78.6%	1/2(NCT03085914)	(Powderly et al. 2022)
	Pembrolizumab Chemotherapy	NSCLC	Epacadostat plus pembrolizumab plus chemotherapy vs. placebo plus pembrolizumab plus chemotherapy	ORR: 26.4% vs. 44.8%; extended response ≥ 6 months: 29.2% vs. 15.4%	TRAEs: 94.4% vs. 89.5%; Grade ≥ 3 TRAEs: 46.7% vs. 41.9%	1/2(NCT03322566)	(Boyer et al. 2024)
BMS-986205	Nivolumab	Advanced Gastric Cancer	BMS-986205 plus Nivolumab	ORR: 13.2%	SAEs: 63.2%	2(NCT02935634)	None
Indoximod (1-methyl-d-tryptophan)	Pembrolizumab	Non-ocular melanoma Stage III Melanoma Stage IV Melanoma	Indoximod plus Pembrolizumab	ORR: 51%; DCR: 70%; mPFS: 12.4%	TRAEs led to discontinuation: 21%	1/2(NCT02073123)	(Zakharia et al. 2021)

AE: Adverse Events; ARG: Arginase; BTC: Biliary Tract Cancer; CRC: Colorectal Cancer; DCR: Disease Control Rate; GLS: Glutaminase; IDO1: Indoleamine 2,3-Dioxygenase 1; mPFS: Median Progression-Free Survival; NSCLC: Non-Small Cell Lung Cancer; ORR: Objective Response Rate; RCC: Renal Cell Carcinoma; SAEs: Serious Adverse Events; TEAEs: Treatment-Emergent Adverse Events; TRAEs: Treatment-Related Adverse Events; UC: Urothelial Cancer

### Glutamine

Telaglenastat (CB-839) is a selective inhibitor of GLS, which blocks the conversion of glutamine to glutamate. A Phase I study assessed the efficacy of telaglenastat in patients with treatment-refractory solid tumors, reporting a disease control rate (DCR) of 43%. Notably, in patients with renal cell carcinoma (RCC), the DCR reached 50% (Harding et al. 2021). Fatigue (23%) and nausea (19%) were the most common adverse events (Harding et al. 2021). Further research has demonstrated the efficacy of telaglenastat in combination with everolimus, an mTOR inhibitor in patients with advanced or metastatic RCC previously treated with tyrosine kinase inhibitors (TKIs) and checkpoint inhibitors. The study reported a median progression-free survival (mPFS) of 3.8 months for the combination of telaglenastat and everolimus, compared to 1.9 months for those treated with placebo plus everolimus (Lee et al. 2022). However, telaglenastat fails to improve the efficacy of cabozantinib, a VEGFR2/MET/AXL inhibitor, in metastatic RCC (Tannir et al. 2022).

### Arginine

INCB001158, an oral arginase inhibitor, has been investigated for its therapeutic efficacy across various clinical settings (see Table 1). Kuboki et al. reported that INCB001158 does not exhibit significant activity as a monotherapy (Kuboki et al. 2024). However, its clinical potential is enhanced when combined with other therapeutic agents. Notably, when administered with pembrolizumab, INCB001158 achieved three partial responses (PRs) and a DCR of 30% among 43 patients with microsatellite-stable colorectal cancer (Naing et al. 2019). Furthermore, INCB001158 has also been explored in combination with gemcitabine/cisplatin in a phase I/II study for patients with advanced biliary tract cancer, demonstrating an overall response rate (ORR) of 24% and stable disease (SD) in 42% of patients (Javle et al. 2021). Adverse events (AEs) associated with INCB001158 and/or chemotherapy occurred in 88% of patients but did not result in significant toxicity (Javle et al. 2021).

### Tryptophan

Epacadostat is a potent and highly selective inhibitor of the IDO1 enzyme. Numerous clinical trials evaluating the combination of Epacadostat with other drugs have reported results (see Table 1). A phase 1/2 trial assessed the combination of epacadostat and pembrolizumab, a programmed death protein-1 (PD-1) inhibitor, in patients with advanced solid tumors. Objective responses were observed in 12 (55%) of 22 patients with specific types of cancer (Mitchell et al. 2018). Indoximod is a small-molecule IDO pathway inhibitor. A Phase 2 trial investigated indoximod in combination with pembrolizumab for the treatment of advanced melanoma. The ORR for the evaluable population was 51%, with a confirmed complete response rate of 20% and a disease control rate of 70%. The mPFS was 12.4 months. The ORR for Programmed Death-Ligand 1 (PD-L1)-positive patients was 70%, compared to 46% for PD-L1-negative patients (Zakharia et al. 2021). In addition, a Phase I/II clinical trial showed that the combination of nivolumab, a PD-1 inhibitor, and IO102/IO103, a first-in-class immune-modulatory vaccine against IDO, resulted in an ORR of 80% and complete responses (CR) of 43% (Kjeldsen et al. 2021). However, the combination of epacadostat and pembrolizumab failed to show efficacy in a Phase 3 trial for patients with unresectable or metastatic melanoma (Long et al. 2019). Epacadostat (100 mg twice daily) plus pembrolizumab did not improve PFS or overall survival compared with placebo plus pembrolizumab (Long et al. 2019). Moreover, in patients with ERBB2-negative metastatic breast cancer, adding indoximod to a taxane did not improve PFS compared to a taxane alone (Mariotti et al. 2021). Odunsi et al. finds that IDO1 inhibition blocks the kynurenine pathway but redirects tryptophan catabolism toward the serotonin pathway. This shift results in elevated levels of nicotinamide adenine dinucleotide (NAD^+^), which subsequently reduces T cell proliferation and function (Odunsi et al. 2022). This finding explains why targeting a single metabolic pathway may fail to produce effective clinical outcomes due to redundancy and feedback adaptation. It suggests that future strategies might need to target multiple metabolic pathways to enhance clinical efficacy.

## Conclusions

Amino acid metabolism plays a pivotal role in regulating myeloid cell functions through both metabolic reprogramming and epigenetic modifications, thereby influencing immune responses and disease progression. Additionally, certain metabolic enzymes, such as PHGDH, have been shown to modulate myeloid cell functions through non-metabolic pathways. Thus, targeting amino acid metabolism emerges as a promising strategy to modulate myeloid cell activity.

In the tumor microenvironment, myeloid cells impair T cell functions by depleting essential amino acids like arginine, tryptophan, and cysteine, or by producing inhibitory metabolites, thereby promoting tumor growth and metastasis. This offers a new perspective on the complex suppressive immune microenvironment and sheds light on the poor response of solid tumors to immunotherapy. Furthermore, supplementing amino acids or targeting key enzymes and metabolites in amino acid metabolic pathways has demonstrated potential in reprogramming myeloid cells, reducing their immunosuppressive functions while enhancing their immune effector functions, ultimately inhibiting tumor growth. These findings suggest novel strategies to enhance the efficacy of immunotherapy.

Despite significant progress, several challenges remain. First, individual amino acids can have opposing effects on myeloid cells. For instance, serine metabolism has been shown to promote both M1 and M2 polarization, suggesting the presence of counteracting mechanisms that remain either undiscovered or not fully understood. Second, current research predominantly focuses on amino acids such as arginine, tryptophan, serine, and glutamine, leaving the effects of other amino acids on myeloid cells largely unexplored. This gap warrants further investigation. Third, clinical studies evaluating drugs that specifically target amino acid metabolism are limited. Most clinical trials involve combinations with anti-PD-1, anti-PD-L1, or chemotherapy agents. Although targeting amino acid metabolism has shown promise in cellular and preclinical models, translating these findings into clinical practice and enhancing the efficacy of current immunotherapies and chemotherapies remains a challenge.

The amino acid metabolic profiling of myeloid cells is still incomplete. Future research should leverage advanced technologies such as metabolomics, single-cell sequencing, and single-cell metabolomics to comprehensively map the metabolic landscapes of myeloid cells. This approach would elucidate the dynamic changes in amino acid metabolism within each myeloid cell type, revealing metabolic heterogeneity both among different myeloid cell subsets and within the same cell type under varying functional states. Such insights could link specific metabolic pathways to critical cellular functions, including inflammation, antigen presentation, and phagocytosis, potentially identifying novel cancer-related biomarkers or metabolic enzymes, thereby offering new targets for cancer immunotherapy. Wu et al. utilized single-cell technologies to profile neutrophils across different tumor types and identified ten distinct phenotypes. Among these, a subset of neutrophils with antitumor effects enhanced their antigen-presenting capability through leucine metabolism (Wu et al. 2024). This finding provides evidence for the role of amino acid metabolism in regulating plasticity of myeloid cells. A comprehensive map of amino acid metabolism in myeloid cells would offer new perspectives on the diverse phenotypes of myeloid cells within the tumor microenvironment and serve as a powerful tool for enhancing immunotherapy strategies.

## Data Availability

No datasets were generated or analysed during the current study.

## Abbreviations

3PHP: 3-phosphohydroxypyruvate
3PG: 3-phosphoglycerate
3PS: 3-phosphoserine
α-KG: Alpha-ketoglutarate
Arg1: Arginase 1
ATF4: Activating transcription factor 4
CR: Complete responses
DCs: Dendritic cells
DCR: Disease control rate
eIF2α: Eukaryotic translation initiation factor 2 alpha
eIF4F: Eukaryotic translation initiation factor 4 F
FAO: Fatty acid oxidation
GCN2: General control nonderepressible 2
GSH: Glutathione
HIF-1α: Hypoxia-inducible factor-1α
IDO1: Indoleamine 2,3-dioxygenase 1
IFN-I: Type I interferon
IL-1β: Interleukin-1β
MDSCs: Myeloid-derived suppressor cells
M-MDSCs: Monocytic MDSCs
mPFS: Median progression-free survival
mTORC1: Mechanistic target of rapamycin complex 1
NETs: Neutrophil extracellular traps
NO: Nitric oxide
NOS: Nitric oxide synthase
ORR: Overall response rate
OXPHOS: Oxidative phosphorylation
PD-L1: Programmed Death-Ligand 1
PHGDH: Phosphoglycerate dehydrogenase
PMN-MDSCs: Polymorphonuclear MDSCs
PPP: Pentose phosphate pathway
PRs: Partial responses
PSAT1: Phosphoserine aminotransferase 1
PSPH: Phosphoserine phosphatase
ROS: Reactive oxygen species
SAM: S-adenosylmethionine
TAMs: Tumor-associated macrophages
TGF-β: Transforming growth factor-β
TLR: Toll-like receptor
TME: Tumor microenvironment
TNF-α: Tumor necrosis factor-α
Tregs: Regulatory T cells
